# Temperature Decline in Embryological Culture Dishes outside Incubator

**DOI:** 10.22074/ijfs.2020.5917

**Published:** 2020-02-25

**Authors:** Dimitra Korakaki, Spyros Mouroutsos, Gregory Tripsianis, Nikos Nikolettos, Byron Asimakopoulos

**Affiliations:** 1 Laboratory of Physiology, Faculty of Medicine, School of Health Sciences, Democritus University of Thrace, Alexandroupolis, Greece; 2 Laboratory of Mechatronics and Systems Automation, Department of Electrical and Computer Engineering, Democritus University of Thrace, Xanthi, Greece; 3 Laboratory of Medical Statistics, Faculty of Medicine, School of Health Sciences, Democritus University of Thrace, Alexandroupolis, Greece

**Keywords:** Culture Dish, Embryo Culture, Temperature

## Abstract

**Background:**

In embryological culture dishes, there is a temperature decline when they are removed outside incubators. This study aimed at investigating the effects of this temperature decline within a certain time frame, the type of culture dish with or without the use of laminar air flow and whether it is possible to achieve a sufficient thermal control with the use of a heating stage.

**Materials and Methods:**

In this experimental study, the temperatures of four different types of polystyrene dishes [50 mm intracytoplasmic sperm injection (ICSI), 35 mm, 60 mm, 90 mm], filled with culture medium and oil were recorded for a period of 10 minutes outside the incubator. Temperature was measured with an infrared thermographic camera. The reference temperature was 37°C. Four parameters were analyzed: the type of dishes, air flow, a heating stage at 37°C and 38.5°C.

**Results:**

There was a time-dependant significant temperature decline outside the incubator in all types of dishes and under all experimental conditions. Under air flow temperature decline increased compared to the no air flow condition. The use of a heating stage at either 37°C or 38.5°C slightly improved the situation in most cases. After three minutes out of the incubator without a heating stage and air flow, the temperature was <34°C; with air flow and without a heat- ing stage the temperature was <33°C. When a heating stage was used, the temperature was <36°C, except when using ICSI dishes. When ICSI dishes were on a heating stage they maintained a temperature close to 37°C with or without air flow. In all experimental conditions the highest decline was recorded with the 90 mm dishes.

**Conclusion:**

Time is crucial for managing the temperature decline in culture dishes when out of the incubator. Under air laminar flow, the heat loss is greater, when with a heating stage at 37°C or better at 38.5°C this loss decreases but still exists. ICSI flat bottom dishes give the best results when heated stages are used. Flat bottom dishes maintain the temperature rather efficiently. Based on our findings, the use of flat bottom dishes should become a universal practice in *in vitro* fertilization (IVF).

## Introduction

Temperature is a crucial factor for *in vitro* cell culture, particularly for *in vitro* culture of human gametes and embryos. The human core body temperature, 37°C, is considered optimal for *in vitro* culture of human gametes and embryos although several studies have shown that the temperature in ovaries and fallopian tubes is lower (1-5). However, attempts to culture human embryos in lower temperatures than 37°C have not given satisfactory results (6, 7). Moreover, experiments with oocytes of both domestic animals and humans have shown that exposure to low temperatures has an impact on spindle integrity, chromosomal organization and fertilization (8-11). Incubators effectively maintain a constant temperature of 37°C, although the temperature inside the incubators may be affected by the frequency of door openings and incubator type.

In the every-day clinical practice, gametes and embryos are routinely exposed to room temperature: during oocyte retrieval, preparation for *in vitro* fertilization (IVF), intracytoplasmic sperm injection (ICSI), assessment of embryo development and embryo transfers. In all of these cases, the temperature decreases despite the fact that embryologists emphasize on minimizing this decrease by reducing the time outside of incubators and by using heating stages. The magnitude of the temperature drop is an issue of concern but has not been studied in detail.

In the present study we have investigated the following questions regarding temperature decline in embryological culture dishes outside incubators: how much is the reduction of the temperature in a certain time frame and type of dish? Is it possible to achieve a sufficient thermal control with the use of heating stages? How much does the laminar air flow affect the temperature?

## Materials and Methods

This was an experimental study, conducted in the Laboratory of Physiology, Faculty of Medicine, School of Health Sciences, Democritus University of Thrace, Alexandroupolis, Greece, from April to June 2018. In this study, no human gametes, embryos, body fluid samples or personal data were utilized.

### Experimental design

Four different types of polystyrene dishes (Nunc IVF Petri Dish; Thermo Scientific, Roskilde, Denmark), were used. All dishes were prepared exactly as if for embryo culture in order to be as close as possible to the working conditions of an embryological laboratory:

- 50 mm ICSI dishes with 5 droplets of culture medium (4 μl each) and a line of 10 μl polyvinyl pyrrolidone (PVP), with 4 ml of oil (group 1).
- 35 mm dishes with 5 droplets of 50 μl culture medium, covered with 4 ml of light mineral oil (group 2).
- 60 mm dishes with 5 droplets of 50 μl culture medium, covered with 12 ml of light mineral oil (group 3).
- 90 mm dishes containing 14 ml of culture medium, without oil (group 4).

Universal IVF Medium (Origio-Sage, Måløv, Denmark) was used for culture medium. Oil for Tissue Culture (Origio-Sage, Måløv, Denmark) was used to overlay the medium droplets. PVP in ICSI dishes was purchased from Origio-Sage (Måløv, Denmark). All dishes were prepared in a vertical laminar flow cabinet Class II (Type A2, ECSO) and then incubated overnight in a Hera Cell 150 incubator (Thermo Electron Co., Germany) set at 37°C. In order to replicate the working conditions in an embryological laboratory, the experimental procedure involved removal of culture dishes from the incubator, abolition of the protective lids of the dishes and recording the temperature of the culture dishes under six experimental conditions: i. On a stereomicroscope without air flow, ii. On a stereomicroscope under vertical laminar air flow, iii. On a microscope with a heating stage set at 37°C without air flow, iv. On a microscope with a heating stage set at 38.5°C without air flow, v. On a microscope with a heating stage set at 37°C under vertical laminar air flow, vi. On a microscope with a heating stage set at 38.5°C under vertical laminar air flow.

For vertical laminar air flow, a class II cabinet (type A2, ECSO) was used with inflow 0.45 m/second and downflow 0.36 m/second. The heating stage used was a MATS-U505R (Tokai Hit Co, LTD, Japan); the dishes were always placed in the centre of the heating stage. The room temperature was monitored at 22°C. Each experiment was repeated 20 times. Overall, twenty four experimental groups were formed:

Group 1: no laminar air flow, no heating
Group 2: no laminar air flow, no heating
Group 3: no laminar air flow, no heating
Group 4: no laminar air flow, no heating
Group 1 air: laminar air flow, no heating
Group 2 air: laminar air flow, no heating
Group 3 air: laminar air flow, no heating
Group 4 air: laminar air flow, no heating
Group 1-37: no laminar air flow, heating at 37°C
Group 2-37: no laminar air flow, heating at 37°C
Group 3-37: no laminar air flow, heating at 37°C
Group 4-37: no laminar air flow, heating at 37°C
Group 1-38.5: no laminar air flow, heating at 38.5°C
Group 2-38.5: no laminar air flow, heating at 38.5°C
Group 3-38.5: no laminar air flow, heating at 38.5°C
Group 4-38.5: no laminar air flow, heating at 38.5°C
Group 1 air 37: laminar air flow, heating at 37°C
Group 2 air 37: laminar air flow, heating at 37°C
Group 3 air 37: laminar air flow, heating at 37°C
Group 4 air 37: laminar air flow, heating at 37°C
Group 1 air 38.5: laminar air flow, heating at 38.5°C
Group 2 air 38.5: laminar air flow, heating at 38.5°C
Group 3 air 38.5: laminar air flow, heating at 38.5°C
Group 4 air 38.5: laminar air flow, heating at 38.5°C

### Temperature assessment

Temperature was measured with a high precision infrared thermographic camera (OPTRIS PI4500, Germany) having thermal sensitivity of 40 mK and optical resolution of 382X288 pixels. The thermographic camera was calibrated by the manufacturer (OPTRIS GmbH, Germany). Additionally, its precision was verified against a resistance temperature detector (Digi-Sense Traceable Memory-Los Model 6442, Cole-Parmer, IL, USA) in the Laboratory of Mechatronics and Systems Automation (Democritus University of Thrace). The camera was connected to a computer allowing continuous recording of temperature. During recordings the camera was always set at the same fixed angle and distance (20 cm) from culture dishes. For statistical analysis we used the recorded temperatures 3, 5 and 10 minutes after removing the culture dishes out of the incubator.

### Statistical analysis

Data was analyzed using the Statistical Package for Social Sciences (SPSS), version 19.0 (IBM, NY, USA). The normality of quantitative variables was tested by Kolmogorov-Smirnov test. All parameters were expressed as mean ± standard deviation (SD). Within groups, differences of quantitative variables were examined by one-way repeated measures analysis of variance (rmANOVA) and post h°C analysis was performed using Sidak’s test. Amongst groups differences at each time point were assessed by one-way ANOVA and Tukey’s test was used for multiple comparisons. The interaction group x time was established by a two-way ANOVA. All tests were two tailed and P<0.05 were considered statistically significant.

## Results

The use of a thermographic camera proved to be very convenient as it measures temperature from a distance (20 cm) allowing for unobstructed manipulation of the culture dishes. The temperature of the culture dishes, as expected, declined significantly after 3, 5 and 10 minutes outside the incubator. The decline depended on the time, the experimental conditions and the type of dish. Working under air flow worsened the decline of temperature and, in general, larger dishes lost temperature faster than the smaller ones. The detailed results are presented in Tables 1-6.

**Table 1:** Temperatures recorded without a heating stage and air laminar flow


Group	Time			
	Onset	3 minutes	5 minutes	10 minutes

1	36.98 ± 0.01^A^	31.30 ± 0.21	30.89 ± 0.18	30.45 ± 0.07^B^
2	36.98 ± 0.01^A^	32.58 ± 0.21	31.51 ± 0.16	30.47 ± 0.09^B^
3	36.98 ± 0.01^A^	33.57 ± 0.13	32.54 ± 0.18	31.16 ± 0.08
4	36.98 ± 0.01^A^	28.56 ± 0.13	27.59 ± 0.19	26.78 ± 0.23


Data are presented as mean ± SD. In each type of culture dish, the differences among the recorded temperatures at different time points were statistically significant (Sidak’s test). In pair-wise comparisons between groups (Tukey’s test) all differences were statistically significant except the cases denoted with the same capital letters. Group 1; 50 mm ICSI dishes with 5 droplets of culture medium (4 μl each) and a line of 10 μl PVP, with 4 ml of oil, Group 2; 35 mm dishes with 5 droplets of 50 μl culture medium, covered with 4 ml of light mineral oil, Group 3; 60 mm dishes with 5 droplets of 50 μl culture medium, covered with 12 ml of light mineral oil, Group 4; 90 mm dishes containing 14 ml of culture medium, without oil. ICSI; Intracytoplasmic sperm injection and PVP; Polyvinyl pyrrolidone.

**Table 2:** Temperatures recorded with laminar air flow and without a heating stage


Group	Time			
	Onset	3 minutes	5 minutes	10 minutes

1 air	36.98 ± 0.01^A^	28.95 ± 0.11	28.33 ± 0.13^B^	27.95 ± 0.05
2 air	36.97 ± 0.01^A^	29.89 ± 0.27	28.18 ± 0.19^B^	26.75 ± 0.07
3 air	36.98 ± 0.01^A^	31.93 ± 0.13	30.46 ± 0.15	28.77 ± 0.07
4 air	36.98 ± 0.01^A^	23.62 ± 0.13	23.05 ± 0.19	22.42 ± 0.08


Data are presented as mean ± SD. In each type of culture dish, the temperature differences recorded at different time points were statistically significant (Sidak’s test). In pair-wise comparisons between groups (Tukey’s test) all differences were statistically significant except the cases denoted with the same capital letters. Group 1 air; 50 mm ICSI dishes with 5 droplets of culture medium (4 μl each) and a line of 10 μl PVP, with 4 ml of oil, Group 2 air; 35 mm dishes with 5 droplets of 50 μl culture medium, covered with 4 ml of light mineral oil, Group 3 air; 60 mm dishes with 5 droplets of 50 μl culture medium, covered with 12 ml of light mineral oil; Group 4 air; 90 mm dishes containing 14 ml of culture medium, without oil. ICSI; Intracytoplasmic sperm injection and PVP; Polyvinyl pyrrolidone.

**Table 3:** Temperatures recorded with a heating stage at 37°C, without air laminar flow


Group	Time			
	Onset	3 minutes	5 minutes	10 minutes

1-37	36.98 ± 0.01^A ^	35.92 ± 0.07	35.62 ± 0.17^*^	35.38 ± 0.20^*^
2-37	36.99 ± 0.01^A^	35.12 ± 0.16	34.61 ± 0.24^B^	33.53 ± 0.12
3-37	36.99 ± 0.01^A^	34.83 ± 0.26^*^	34.48 ± 0.42^B* ^	33.80 ± 0.05
4-37	36.98 ± 0.01^A^	30.79 ± 0.15	29.48 ± 0.21^B^	28.37 ± 0.10


Data are presented as mean ± SD. In each type of culture dish, the temperature differences recorded at different time points were statistically significant except the cases denoted with *(Sidak’s test). In pair-wise comparisons between groups (Tukey’s test) all differences were statistically significant except the cases denoted with the same capital letters. Group 1-37; 50 mm ICSI dishes with 5 droplets of culture medium (4 μl each) and a line of 10 μl PVP, with 4 ml of oil, Group 2-37; 35 mm dishes with 5 droplets of 50 μl culture medium, covered with 4 ml of light mineral oil, Group 3-37; 60 mm dishes with 5 droplets of 50 μl culture medium, covered with 12 ml of light mineral oil, Group 4-37; 90 mm dishes containing 14 ml of culture medium, without oil. ICSI; Intracytoplasmic sperm injection and PVP; Polyvinyl pyrrolidone.

**Table 4:** Temperatures recorded with heating stage at 37°C and air laminar flow


Group	Time			
	Onset	3 minutes	5 minutes	10 minutes

1 air 37	36.98 ± 0.01^A^	34.68 ± 0.08	34.48 ± 0.15	34.18 ± 0.10
2 air 37	36.98 ± 0.01^A^	33.91 ± 0.07	33.23 ± 0.07	32.77 ± 0.07
3 air 37	36.99 ± 0.01^A^	34.46 ± 0.08	33.79 ± 0.07	32.94 ± 0.02
4 air 37	36.98 ± 0.01^A^	27.78 ± 0.20	26.42 ± 0.22	25.34 ± 0.20


Data are presented as mean ± SD. In each type of culture dish, the temperature differences recorded at different time points were statistically significant (Sidak’s test). In pair-wise comparisons between groups (Tukey’s test) all differences were statistically significant except the cases denoted with the same capital letters. Group 1 air 37; 50 mm ICSI dishes with 5 droplets of culture medium (4 μl each) and a line of 10 μl PVP, with 4 ml of oil, Group 2 air 37; 35 mm dishes with 5 droplets of 50 μl culture medium, covered with 4 ml of light mineral oil, Group 3 air 37; 60 mm dishes with 5 droplets of 50 μl culture medium, covered with 12 ml of light mineral oil, Group 4 air 37; 90 mm dishes containing 14 ml of culture medium, without oil. ICSI; Intracytoplasmic sperm injection and PVP; Polyvinyl pyrrolidone.

**Table 5:** Temperatures recorded with the heating stage at 38.5°C, without laminar air flow


Group	Time			
	Onset	3 minutes	5 minutes	10 minutes

1-38.5	36.98 ± 0.01^A^	36.71 ± 0.17^*^	36.77 ± 0.16^*^	36.65 ± 0.11^*^
2-38.5	36.99 ± 0.01^A^	35.65 ± 0.24	35.31 ± 0.20	34.63 ± 0.12
3-38.5	36.98 ± 0.01^A^	36.20 ± 0.21^*^	36.19 ± 0.21^* ^	35.86 ± 0.07
4-38.5	36.98 ± 0.01^A^	31.48 ± 0.29	30.36 ± 0.20	29.47 ± 0.07


Data are presented as mean ± SD. In each type of culture dish, the temperature differences recorded at different time points were statistically significant (Sidak’s test) except the cases denoted with *. In pair-wise comparisons between groups (Tukey’s test) all differences were statistically significant except the cases denoted with the same capital letters. Group 1-38.5; 50 mm ICSI dishes with 5 droplets of culture medium (4 μl each) and a line of 10 μl PVP, with 4ml of oil, Group 2-38.5; 35 mm dishes with 5 droplets of 50 μl culture medium, covered with 4 ml of light mineral oil, Group 3-38.5; 60 mm dishes with 5 droplets of 50 μl culture medium, covered with 12 ml of light mineral oil, Group 4-38.5; 90 mm dishes containing 14 ml of culture medium, without oil. ICSI; Intracytoplasmic sperm injection and PVP; Polyvinyl pyrrolidone.

**Table 6:** Temperatures recorded with the heating stage at 38.5°C and air laminar flow


Group	Time			
	Onset	3 minutes	5 minutes	10 minutes

1 air 38.5	36.99 ± 0.01^A^	36.32 ± 0.11^*^	36.20 ± 0.12^* ^	36.00 ± 0.04
2 air 38.5	36.99 ± 0.01^A^	35.65 ± 0.10	35.20 ± 0.11	34.73 ± 0.04
3 air 38.5	36.99 ± 0.01^A^	35.36 ± 0.15	34.85 ± 0.16	34.03 ± 0.22
4 air 38.5	36.99 ± 0.01^A^	28.75 ± 0.13	27.85 ± 0.16	27.56 ± 0.08


Data are presented as mean ± SD. In each type of culture dish, the temperature differences recorded at different time points were statistically significant (Sidak’s test) except the cases denoted with *. In pair-wise comparisons between groups (Tukey’s test) all differences were statistically significant except the cases denoted with the same capital letters. Group 1 air 38.5; 50 mm ICSI dishes with 5 droplets of culture medium (4 μl each) and a line of 10 μl PVP, with 4 ml of oil, Group 2 air 38.5; 35 mm dishes with 5 droplets of 50 μl culture medium, covered with 4 ml of light mineral oil, Group 3 air 38.5; 60 mm dishes with 5 droplets of 50 μl culture medium, covered with 12 ml of light mineral oil, Group 4 air 38.5; 90 mm dishes containing 14 ml of culture medium, without oil. ICSI; Intracytoplasmic sperm injection and PVP; Polyvinyl pyrrolidone.

Overall, the highest reductions of temperature were recorded with 90 mm culture dishes. There was a dramatic reduction of temperature under air flow, without heating; 3 minutes after removal from the incubator, the temperature was 23.62 ± 0.13°C and at 10 minutes it had almost reached the room temperature (22.42 ± 0.08°C).

When the heating stage was on, the lowest reductions were recorded with 50 mm ICSI dishes. After 3 minutes working on a heating stage at 37°C and without air flow, ICSI dishes reached 35.92 ± 0.17°C and remained above 35°C for the rest of the time until 10 minutes. At three time points on a heating stage working at 38.5°C and without air flow, ICSI dishes reached 36.71 ± 0.17°C and remained above 36°C up to 10 minutes. When there was air flow and the heating stage was at 38.5oC, ICSI dishes maintained a temperature of 36.32 ± 0.11°C at 3 minutes and 36.00 ± 0.04°C at 10 minutes outside the incubator.

Instead, when the heating stage was off, the lowest reductions were observed in 35 mm culture dishes, although in all cases they failed to maintain a temperature close to 37°C.

## Discussion

The results of this study highlight the significance of time, stage conditions and most importantly the type of culture dishes. Regarding the time, it is obvious that a decline of temperature always happens when moving the dishes outside the incubator, but this decline becomes dramatic and detrimental for gametes and embryos as this time increases. In general, keeping the culture dishes for 5 to 10 minutes outside the incubator, even on a heating stage, should be avoided. During all the experiments, the room temperature was constantly in 22°C, nonetheless, we can speculate that even at a higher room temperature (e.g. 24°C) the decline of temperature in the culture dishes would have been similar, perhaps only slightly less significant.

Laminar air flow is another factor in increasing the speed of heat loss in culture dishes. However, working under laminar air flow, especially vertical air flow with class II cabinets, is essential for providing a clean and safe environment. Consequently, although the temperature decline increases in the presence of air flow, working without air flow is not considered an option in an embryological laboratory.

Heating stages are universally used to maintain the temperature of culture dishes outside the incubator. Here, it is worth underlining that heating stages cannot actually maintain the temperature of culture dishes at 37°C but rather lower the drop of it. According to the results of the present study, heating stages should be set at a temperature higher than 37°C in order to have better results. The exact working temperature should be decided in each laboratory according to their environmental conditions, specifically room temperature and air flow. In any case, a working temperature much higher than 38°C should be avoided even for a short time period, because it may heat the culture dishes above 37°C. Several studies have shown that temperatures higher than the core body temperature are more detrimental to embryos than temperatures lower than the core body temperature (12-14). In this study, by setting the heating stage at 38.5°C, we did not observe even a transient temperature increase above 37°C in the culture dishes.

The type of dishes is another factor influencing the temperature decline. For instance, 90 mm dishes showed the highest temperature decline in all experimental conditions. On the other hand, ICSI dishes had the best results when the heating stage was in use. Under laminar flow and with the heated stage at 38.5°C, ICSI dishes maintained a temperature of 36.32 ± 0.11°C, 36.20 ± 0.12°C and 36.00 ± 0.04°C at 3, 5 and 10 minutes outside the incubator respectively. It is worth mentioning that according to previous studies (6-7), 36-36.5°C is considered as an acceptable temperature for oocytes and embryos and while it does not provide any benefits, it seems that it does not cause any harms either. In our opinion, the better results associated with the ICSI dish are explained, at least partially, by its flat bottom. All the other types of dishes have a rim around the bottom that does not permit the bottom to touch the heating stage directly. For this reason, heat transfer is prevented by a thin air layer between the heating stage and the bottom of the culture dish. ICSI dishes with flat bottom are heated more effectively when placed on a heated stage and consequently maintain a safe temperature better than other types of dishes outside the incubator. It is strange why most IVF dishes are manufactured with a rim around the bottom. This is probably due to the safety issues in cell culture practice, as in cell culture dishes are usually stacked, one on top of the other, in the incubators and the rim around the dishes helps fix them on each other so they do not fall out easily. This practice, however, is not the case in most embryological laboratories.

## Conclusion

The present study shows that time is a crucial factor for temperature decline in culture dishes outside the incubator. Keeping the culture dishes outside the incubator for more than 3 minutes results in a dramatic decline of temperature. The golden rule here is: the quicker the better. Under air flow, the heat loss is greater and on a heating stage at 37°C or better at 38.5°C the loss is lower but still exists. The loss of heat is influenced by the type of dish as well. The best results, when heated stages were used, were recorded when flat bottom dishes were tested. Flat bottom dishes are heated effectively and maintain the temperature efficiently in a short time period. Based on our findings, the use of flat bottom dishes should become universal in IVF.

## References

[1] Grinsted J, Blendstrup K, Andreasen MP, Byskov AG (1980). Temperature measurements of rabbit antral follicles. J Reprod Fertil.

[2] Hunter RH, Nichol R (1986). A preovulatory temperature gradient between the isthmus and ampulla of pig oviducts during the phase of sperm storage. J Reprod Fert.

[3] Bahat A, Eisenbach M, Tur-Kapsa I (2005). Periovulatory increase in temperature difference within the rabbit oviduct. Hum Reprod.

[4] Baak NA, Cantineau AEP, Farquhar C, Brison D (2016). Temperature of embryo culture for assisted reproduction. Cochrane Database of Systematic Reviews.

[5] Ng KYB, Mingels R, Morgan H, Macklon N, Cheong Y (2018). In vivo oxygen, temperature and pH dynamics in the female reproductive tract and their importance in the human conception: a systematic review. Hum Reprod Update.

[6] Hong KH, Lee H, Forman EJ, Upham KM, Scott RT Jr (2014). Examining the temperature of embryo culture in in vitro fertilization: a randomized controlled trial comparing traditional core temperature (37oC) to a more physiologic, cooler temperature (36°C). Fertil Steril.

[7] Fawzy M, Emad M, Gad MA, Sabry M, Kasem H, Mahmoud M, Bedaiwy MA (2018). Comparing 36,5°C with 37°C for human embryo culture: a prospective randomized controlled trial. RBM Online.

[8] Hirao Y, Yanagimachi R (1978). Temperature dependence of sperm-egg fusion and post-fusion events in Humster fertilization. J Exp Zool.

[9] Lenz RW, Ball GD, Leibfried ML, Ax RL, First NL (1983). In vitro maturation and fertilization of bovine oocytes are temperature dependent processes. Biol Reprod.

[10] Moor RM, Crosby IM (1985). Temperature-induced abnormalities in sheep oocytes during maturation. J Reprod Fertil.

[11] Almeida PA, Bolton VN (1995). The effect of temperature fluctuations on the cytoskeletal organisation and chromosomal constitution of the human oocyte. Zygote.

[12] Alliston CW, Howarth B Jr, Ulberg LC (1965). Embryonic mortality following culture in-vitro of one- and two-cell rabbit eggs at elevated temperatures. J Reprod Fertil.

[13] Sun XF, Wang WH, Keefe DL (2004). Overheating is detrimental to meiotic spindles within in vitro matured human oocytes. Zygote.

[14] Yamanaka KI, Khatun H, Egashira J, Balboula AZ, Tatemoto H, Sakatani M (2018). Heat-shock-induced cathepsin B activity during IVF and culture compromises the developmental competence of bovine embryos. Theriogenology.

